# Oxidative Phosphorylation System in Gastric Carcinomas and Gastritis

**DOI:** 10.1155/2017/1320241

**Published:** 2017-06-28

**Authors:** René G. Feichtinger, Daniel Neureiter, Tom Skaria, Silja Wessler, Timothy L. Cover, Johannes A. Mayr, Franz A. Zimmermann, Gernot Posselt, Wolfgang Sperl, Barbara Kofler

**Affiliations:** ^1^Laura-Bassi Centre of Expertise, Research Program for Receptor Biochemistry and Tumor Metabolism, Department of Pediatrics, Paracelsus Medical University, 5020 Salzburg, Austria; ^2^Institute of Pathology, Paracelsus Medical University, 5020 Salzburg, Austria; ^3^Division of Molecular Biology, Department of Microbiology, Paris-Lodron University, 5020 Salzburg, Austria; ^4^Department of Medicine and Department of Pathology, Microbiology, and Immunology, Vanderbilt University School of Medicine and Veterans Affairs Tennessee Valley Healthcare System, Nashville, TN 37232, USA; ^5^Department of Pediatrics, Paracelsus Medical University, 5020 Salzburg, Austria

## Abstract

Switching of cellular energy production from oxidative phosphorylation (OXPHOS) by mitochondria to aerobic glycolysis occurs in many types of tumors. However, the significance of this switching for the development of gastric carcinoma and what connection it may have to *Helicobacter pylori* infection of the gut, a primary cause of gastric cancer, are poorly understood. Therefore, we investigated the expression of OXPHOS complexes in two types of human gastric carcinomas (“intestinal” and “diffuse”), bacterial gastritis with and without metaplasia, and chemically induced gastritis by using immunohistochemistry. Furthermore, we analyzed the effect of HP infection on several key mitochondrial proteins. Complex I expression was significantly reduced in intestinal type (but not diffuse) gastric carcinomas compared to adjacent control tissue, and the reduction was independent of HP infection. Significantly, higher complex I and complex II expression was present in large tumors. Furthermore, higher complex II and complex III protein levels were also obvious in grade 3 versus grade 2. No differences of OXPHOS complexes and markers of mitochondrial biogenesis were found between bacterially caused and chemically induced gastritis. Thus, intestinal gastric carcinomas, but not precancerous stages, are frequently characterized by loss of complex I, and this pathophysiology occurs independently of HP infection.

## 1. Introduction

Histologically, two main types of gastric cancers (GCs) can be distinguished according to the Laurén classification [1, 2]. The intestinal type of GC progresses through a well-characterized process of morphological changes: normal mucosa, chronic gastritis, atrophic gastritis, intestinal metaplasia, and cancer. The relative frequencies are approximately 54% for the intestinal type and 32% for the diffuse type, with the remaining 15% of CGs being indeterminate [3]. The prognostic relevance of the Laurén classification is still a matter of debate as some studies found no correlation with patient outcome [4]. Intestinal type GC is most prevalent in older men, whereas the diffuse type is more prevalent in young women [5]. Intestinal type was reported to be associated with intestinal metaplasia of the gastric mucosa and presence of *Helicobacter pylori* (HP) [6].

Most human tumors are characterized by a shift from oxidative phosphorylation (OXPHOS) to aerobic glycolysis called the Warburg effect [7–13]. At least two of the five OXPHOS complexes act as tumor suppressors, namely, complex I in oncocytic tumors and complex II in both pheochromocytomas and paragangliomas [11, 14–17]. In addition, several mutations affecting mitochondrial complex I genes have been described in gastric cancers and other neoplasms, suggesting that mitochondrial energy metabolism may play a critical role in tumor development [18–22]. During the last decade, our understanding of cancer energy metabolism changed fundamentally. It is now known that tumor cells can use glucose and glutamine, the latter is the preferred substrate for OXPHOS. In addition, it is proposed that many cancer types use respiration and glycolysis for energy production [23]. Other models of cancer metabolism like the “reverse Warburg effect” and the “lactate shuttle hypothesis” have emerged reflecting the heterogeneity and flexibility of tumor energy metabolism [24–27].

The most common cause of GC is infection by HP, which is classified as a type 1 (definite) carcinogen for GC by the World Health Organization [28]. The two major virulence factors of HP are vacuolating cytotoxin A (VacA) and cytotoxin-associated gene A (CagA). VacA is excreted into the extracellular space, where it can bind and enter host cells and form channels in the inner mitochondrial membrane, leading to depolarization of mitochondrial membrane potential, disruption of mitochondrial function, and ultimately cell death [29–33]. Infection of AGS (adenocarcinoma gastric cell line) cells with HP leads to mtDNA instability and a decrease in mtDNA copy number [34, 35]. The amount and activity of complex I decreased after infection with HP [36]. VacA can induce the recruitment of dynamin-related protein-1 (DRP-1) to induce mitochondrial network fragmentation [32]. Finally, VacA can induce (acute exposure) or repress (prolonged exposure) autophagy, events which also can potentially influence mitochondrial energy metabolism [37]. Intracellular VacA is significantly associated with the development of progressive atrophic gastritis and intestinal metaplasia [38]. We hypothesized that a large number of gastric carcinomas similar to other solid tumors are characterized by the Warburg effect. Furthermore, we propose that HP infection has an influence on mitochondrial energy metabolism.

The aim of the present study was to elucidate changes in aerobic mitochondrial energy metabolism in GC and gastritis and evaluate the pathophysiologic significance of HP infection on expression of subunits of the OXPHOS complexes. Immunohistochemical staining was used, because it is impossible to get a sufficient amount of frozen tissue for functional evaluation of the OXPHOS enzymes especially in diffuse gastric carcinomas and gastritis. Diffuse GCs grow in relatively small cell clusters interspersed by a large number of normal cells. Intestinal gastric carcinomas also are heterogeneous with regard to tissue composition. In addition, heterogeneity is also present within a single intestinal GC. Furthermore, a tumor cell content of over 80% is needed to generate reliable functional data on the OXPHOS enzyme activity. Immunohistochemical staining of homogenous tissue samples correlates well with enzymatic analysis as the OXPHOS system is mainly regulated via protein amount [7, 10, 11]. Therefore, immunohistochemical staining of heterogeneous samples represents the method of choice since it excellently reflects the in vivo situation.

## 2. Materials and Methods

### 2.1. Ethics

Human tumors were obtained from the Department of Pathology, Paracelsus Medical University, Salzburg. The study was performed according to the Austrian Gene Technology Act. Experiments were conducted in accordance with the Helsinki Declaration of 1975 (revised 1983) and the guidelines of the Salzburg State Ethics Research Committee (ethical agreement: AZ 209-11-E1/823-2006), being no clinical drug trial or epidemiological investigation. All patients signed an informed consent document concerning the surgical intervention. Furthermore, the study did not extend to examination of individual case records. Patient anonymity was ensured at all times. Patient characteristics are given in Supplementary Table 2 available online at https://doi.org/10.1155/2017/1320241.

### 2.2. Samples

Formalin fixed paraffin-embedded (FFPE) tissue from intestinal (*n* = 20) and diffuse-type (*n* = 20) gastric carcinomas, bacterial gastritis (*n* = 5), bacterial gastritis with metaplasia (*n* = 5), and chemical (C) gastritis (*n* = 5) was obtained by the Institute of Pathology Salzburg. Since it is a matter of debate which classification of gastric carcinomas should be used, we added in addition to the Laurén classification also the WHO classification 2010 in Supplementary Tables 2 and 3. All gastric specimens were routinely stained with H&E to obtain the basic morphology of acute or chronic inflammation, fibrosis, and intestinal metaplasia. The presence of metaplasia was confirmed by a periodic acid-Schiff procedure/Alcian blue stain. Additionally, Giemsa staining was performed to visualize *H. pylori*. Based on these detailed histological evaluation patterns, the human gastric biopsies were categorized as chemical gastropathies caused by NSAIDs (nonsteroidal anti-inflammatory drugs) and/or alkaline reflux (in short chemical gastritis) as well as *H. pylori*-associated chronic gastritis with and without intestinal metaplasia (in short, bacterial gastritis).

### 2.3. Immunohistochemical Staining and Analysis of FFPE Tissues

For immunohistochemistry, the following antibodies were used: complex I subunit NDUFS4 (mouse monoclonal, 1 : 1000; Abcam, Cambridge, UK), complex II subunit 70 kDa Fp (mouse monoclonal, 1 : 2000; MitoSciences, Eugene, Oregon), complex III subunit Core 2 (mouse monoclonal, 1 : 1500; MitoSciences), complex IV subunit I (mouse monoclonal, 1 : 1000; MitoSciences), complex V subunit alpha (mouse monoclonal, 1 : 2000; MitoSciences), porin 31HL (mouse monoclonal, 1 : 3000; MitoSciences), and VacA antibody (rabbit polyclonal, 1 : 1000) [39]. All antibodies were diluted in Dako antibody diluent with background reducing components (Dako, Glostrup, Denmark). Immunohistochemistry was performed as described previously [40]. For antigen retrieval, the sections were immersed for 45 min in 1 mM EDTA, 0.05% Tween-20, pH 8, at 95°C. Tissue sections were incubated for 30 min with the above-mentioned primary antibodies. The staining intensities of the tumor and control tissues were determined by two examiners on a stereomicroscope. Staining intensities were rated using a scoring system ranging from 0 to 3, with 0 indicating no staining, 1 mild, 2 moderate, and 3 strong staining. The intensities were multiplied by the percentage of positive cells, resulting in score values.

The specificity of the antibodies was previously shown by Western blot analysis: ([9]; Figure 3), ([8], Figure 2); NDUFS4 ([9]; Figure 3), ([8], Figure 2); SDHA ([9]; Figure 3), ([8], Figure 2); UQCRC2 ([9]; Figure 3), ([8], Figure 2); MT-CO1 ([41], Figure 2); ATP5A ([9]; Figure 3), ([42]; Figure 3).

### 2.4. Immunofluorescence Staining of Gastritis

Cells grown on chamber slides were fixed overnight in neutral-buffered formalin. For antigen retrieval, the sections were immersed for 45 min in 1 mM EDTA, 0.05% Tween-20, pH 8, at 95°C. Tissue sections were incubated for 1 h with the above-mentioned primary antibodies. Slides were incubated with a mixture of donkey anti-mouse AlexaFluor488 (1 : 500; Fisher Scientific) and donkey anti-rabbit AlexaFluor594 (1 : 1000; Fisher Scientific) in PBS-T for 1 h. Nuclei were stained with DAPI for 10 min. Slides were mounted with fluorescence mounting medium (DAKO) and sealed with a nail polish. For immunofluorescence staining, the same antibodies as for immunohistochemical staining were used with the following dilution: porin (rabbit polyclonal; 1 : 400, Abcam), NDUFS4 (1 : 100), SDHA (1 : 400), UQCRC2 (1 : 400), MT-CO1 (1 : 100), ATP5A (1 : 400), TFAM (1 : 100), and VacA (1 : 100).

### 2.5. Determination of mtDNA Mutations

Three 5 *μ*m-thick tissue sections were microdissected to separate normal tissue and tumor tissue. The microdissected regions were used for isolation of DNA. mtDNA copy number and mitochondrial common deletions were determined by quantitative PCR as previously described [43].

In addition, the genes of mitochondrially encoded subunits of complex I and tRNAs were sequenced in samples that showed the most severe loss of complexes I and IV. Primer sequences are given in Supplementary Table 1 that were previously published [8]. Sequencing was performed with a GenomeLab™ DTCS Quick Start Kit (Beckman Coulter, Fullerton, CA) on a CEQ™ 2000 DNA Analysis System (Beckman Coulter). The CEQ 8000 Genetic Analysis System (Beckman Coulter) was used for analysis of the sequencing data. These sequences were compared with the complete *Homo sapiens* mitochondrial genome (GenBank accession number: J01415.1).

### 2.6. Statistical Analysis

Patient characteristics and clinical data were analyzed with the *χ*^2^ test and Student's *t*-test. For multiple comparisons of intestinal and diffuse-type GC and control tissue, one-way ANOVA and Bonferroni correction were used. For analysis of clinical parameters and OXPHOS enzyme expression, *t*-test or ANOVA and Bonferroni post hoc test were used. For analysis of gastritis, the nonparametric Kruskal-Wallis test was used. For comparison of mtDNA copy number in intestinal GC with and without HP history, a *t*-test was used.

## 3. Results

### 3.1. Expression of OXPHOS Complexes and Porin Differs between Intestinal and Diffuse-Type Gastric Carcinomas

**Figure 1:**
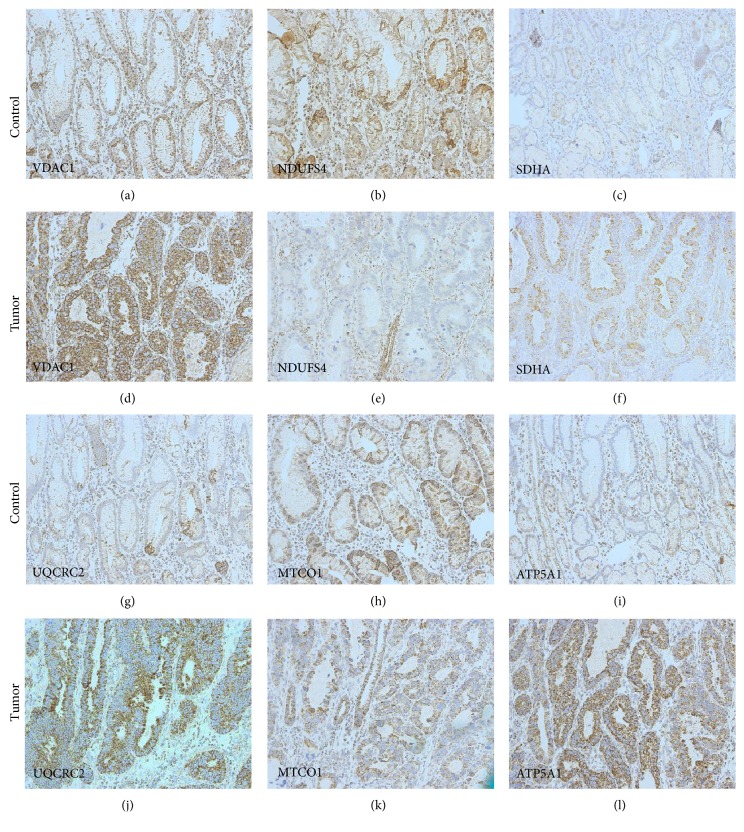
Expression of the OXPHOS complexes and porin in intestinal GC (case M5). Immunohistochemical staining of (a–c); (g–i) normal gastric mucosa and (d–f); (j–l) intestinal gastric carcinoma for (a), (d) porin, (b), (e) complex I, (c), (f) complex II, (g), (j) complex III, (h), (k) complex IV, (i), and (l) complex V. Magnification 20x.

**Figure 2:**
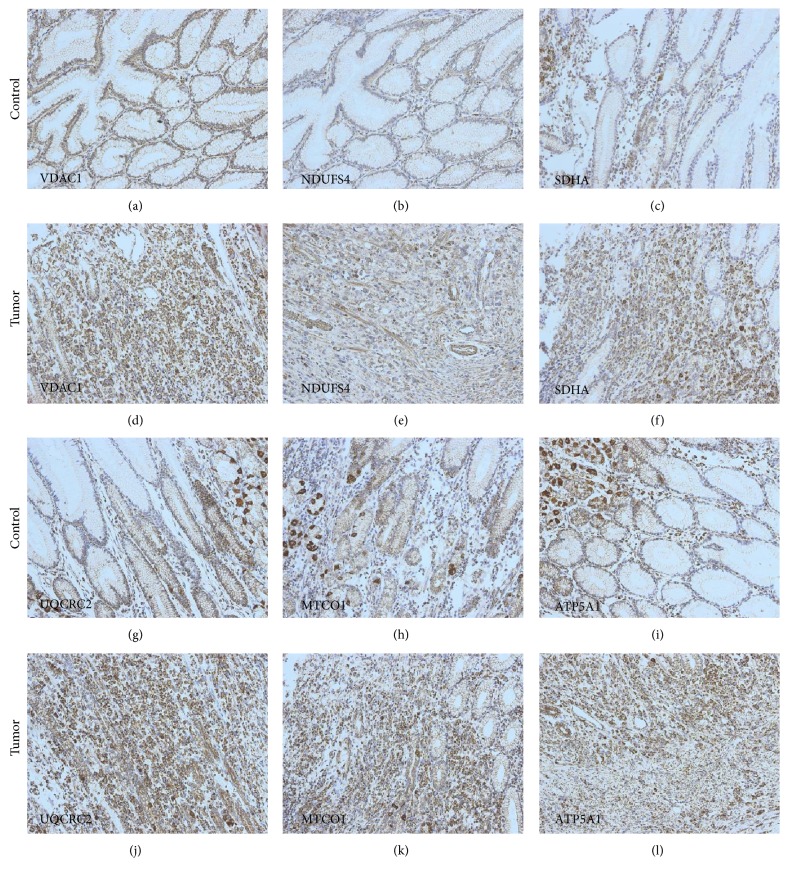
Expression of the OXPHOS complexes and porin in diffuse-type GC (case M39). Immunohistochemical staining of (a–c); (g–i) normal gastric mucosa and (d–f); (j–l) diffuse-type gastric carcinoma for (a), (d) porin, (b), (e) complex I, (c), (f) complex II, (g), (j) complex III, (h), (k) complex IV, (i), and (l) complex V. Parietal cells show strong staining for the OXPHOS complexes (g–i). Magnification 20x.

**Figure 3:**
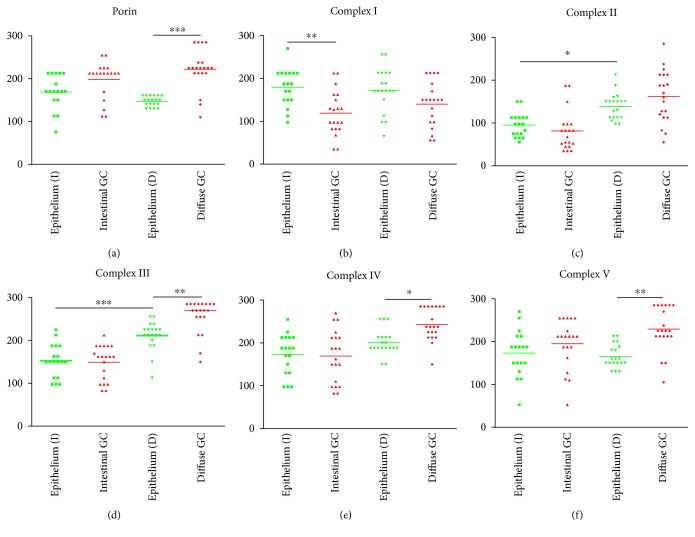
Score values of immunohistochemical staining of the OXPHOS complexes and porin in intestinal and diffuse-type GCs. (a) Porin. (b) Complex I. (c) Complex II. (d) Complex III. (e) Complex IV. (f) Complex V. Tumors were compared to corresponding adjacent control tissue. Corresponding epithelium was used for comparison with intestinal carcinoma I and diffuse-type carcinomas (d). For statistical analysis, a one-way ANOVA with a Bonferroni correction to compare multiple groups was used. ^∗^*p* < 0.05; ^∗∗^*p* < 0.01; ^∗∗∗^*p* < 0.001.

To investigate potential alterations of mitochondrial metabolism in GC, we carried out immunohistochemical staining of the mitochondrial outer membrane protein porin (a marker of mitochondrial mass) as well as all five OXPHOS complexes in tumor samples from 40 patients and compared the staining intensities with those of adjacent normal mucosal columnar epithelial tissues (Figures 1–3; Supplementary Figure 1; Supplementary Tables 4 and 5). We observed significantly higher porin expression in diffuse-type GC compared to adjacent normal tissue (Figures 2(a) and 2(d) and Figure 3(a)). We also detected a trend toward higher porin levels in intestinal GC compared to adjacent normal tissue in 15 of 19 cases examined (Figures 1(a) and 1(d) and Figure 3(a)). Comparison of the seven cases with OXPHOS deficiencies (six complex I and complex IV) revealed that porin, the marker for the mitochondrial mass, is higher in the OXPHOS-deficient cases (mean score value 230) compared to OXPHOS competent cases (mean score value 183).

Complex I staining was significantly reduced in 16 of 20 (80%) intestinal type GCs compared to normal adjacent control tissue (Figures 1(b) and 1(e) and Figure 3(b)). Moreover, although we observed complex I-negative cells in all 20 intestinal GCs, connected large foci of complex I-negative tumor cells were evident in 30% of the cases (35–65% of the tumor cells were negative for complex I in the latter tumors). These cases were also used for microdissection to elucidate the underlying genetic causes (see below).

Overall, complex II showed a trend toward reduced levels in intestinal GCs, as a minor reduction of complex II was present in 16 of 20 (80%) cases compared to adjacent control tissue (Supplementary Figure 1). One case with reduced complex I expression had higher complex II expression (case M17), and one case with unchanged complex I expression had a reduced complex II expression (case M16) compared to normal tissue. In 18 of 20 cases (90%), a combined reduction (*n* = 15) or upregulation (*n* = 3) of complexes I and II was evident. Complex V expression was greatly reduced (by about 50%) in only a single case (case M4); no major differences in complex V were detected in any of the other cases (Supplementary Figure 1). Alterations of complex III and IV were quite heterogeneous, as complex III and complex IV were both higher in 8/20, equal in 3/20, or both lower in 9/20 intestinal GCs.

In the diffuse-type GCs, we detected a trend toward lower expression levels of complex I compared to adjacent control tissue (Figure 1(b)). However, complex I deficiency was rare and found only in a subset of carcinoma cells. Complex III, complex IV, and complex V levels were all significantly higher in diffuse-type GCs compared to normal tissue (Figures 3(d), 3(e), and 3(f); Supplementary Figure 2).

The normal epithelium surrounding intestinal and diffuse-type GCs differed significantly with respect to complexes II and III expression (Figures 3(c) and 3(d); Supplementary Figure 2). This can be explained by the anatomic localization of the biopsy site, since diffuse-type GCs are not associated with the antrum [44], whereas HP-associated intestinal GCs are often found in the antrum [45]. Regional differences in OXPHOS enzyme expression might be present between the fundus, cardia, and antrum.

**Figure 4:**
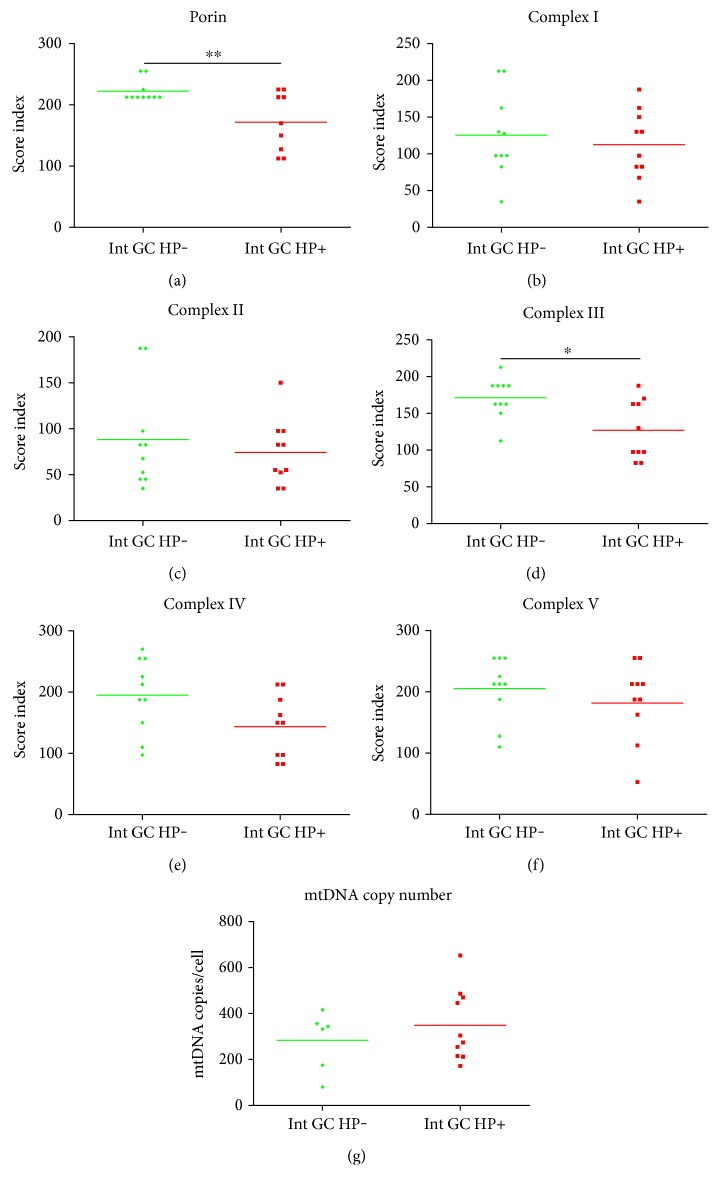
Comparison of score values of immunohistochemical staining of the OXPHOS complexes and porin in intestinal GCs with and without HP history. (a) Porin. (b) Complex I. (c) Complex II. (d) Complex III. (e) Complex IV. (f) Complex V. (g) mtDNA copy number. For statistical analysis, a *t*-test was used. ^∗^*p* < 0.05; ^∗∗^*p* < 0.01.

Of the 20 intestinal GCs, ten had a former histochemical-proven HP infection and we compared them to the ten cases without it. All gastric specimens were routinely stained with H&E to obtain the basic morphology of acute or chronic inflammation, fibrosis, and intestinal metaplasia. The presence of metaplasia was confirmed by a periodic acid-Schiff procedure/Alcian blue stain. Gastric specimens were classified as HP-positive or HP-negative based on the results of the Giemsa stain [46]. We detected significantly lower levels of porin (Figure 4(a)) and complex III (Figure 4(d)) in intestinal GC cases with a history of HP infection. Furthermore, the other complexes also showed a trend toward lower levels in the HP-infected cases. mtDNA copy number was significantly higher in tumors with versus tumors without a history of HP infection. The expression levels of porin and the OXPHOS complexes from normal tissue adjacent to cancerous tissue did not differ between HP-infected and noninfected samples.

### 3.2. Differences in OXPHOS Complex and Porin Expression Related to Clinical Data

Tumors were categorized in low-size (<5 cm) and high-size (>5 cm) tumors by the mean tumor size (5 cm). A significantly higher complex I (*p* = 0.005) and complex II (*p* = 0.011) expression was present in high-size tumors. Furthermore, a higher expression of complex II was found in grade 3 versus grade 2 (*p* = 0.018) and T4 versus T1 (*p* = 0.013). Significantly lower complex III protein levels were found in tumors localized in the prepyloric antrum compared to the cardia (*p* = 0.049). Significantly higher complex III expression was also found in UICC (Union Internationale Contre le Cancer) III compared to UICC II tumors (Supplementary Table 3).

### 3.3. mtDNA Mutations Are Rare in Intestinal Carcinomas

**Figure 5:**
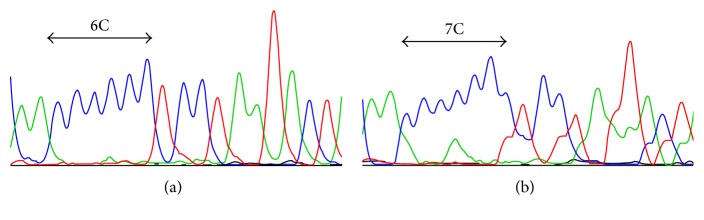
Sequence analysis of complex I coding mtDNA genes in an intestinal GC (case M5). (a) Sequence analysis showing the normal sequence for the poly C stretch of the *ND4* gene from normal corresponding tissue. (b) Sequence analysis showing an insertion of cytosine in the poly cytosine stretch of the *ND4* gene in the tumor tissue. The mutation appears to be heteroplasmic, but the tissue of case M5 was heterogeneous and contaminated with nontumor cells. In addition, complex IV was reduced in M5 (Figures 1(h) and 1(k)).

To investigate if the complex I-negative foci of intestinal GCs are caused by mtDNA mutation, we obtained complex I-negative tissue by microdissection and sequenced selected mtDNA regions, including genes encoding subunits of complex I and tRNAs, from six intestinal GC cases. Only one of the six GCs (case M5; histology shown in Figure 1(e)) harbored a somatic pathogenic mutation, a frame shift (10952_10953insC) in a poly cytosine stretch of the complex I subunit ND4 (Figure 5). This insertion causes a frameshift and creates a stop codon ~ 150 bp downstream, which results in a truncated ND4 protein. The same mutation has also been reported in thyroid oncocytomas [40].

### 3.4. Analysis of Reported Mutations in Nuclear-Encoded Complex I Subunits

The Cancer Genome Project database COSMIC was used to check the frequency of potentially pathogenic nuclear-encoded complex I subunits in diffuse and intestinal gastric carcinomas. The pathogenicity of the mutations was analyzed with MutationTaster. In total, 8 potentially pathogenic mutations were identified in 85 analyzed intestinal adenocarcinomas. Mutations included 2 frame shift mutations, one stop mutation and five missense mutations. In 9.4% of the adenocarcinomas, mutations were identified. This could only partially explain the high frequency of complex I deficiency as found in our study that was 35%. However, since the tumors often show a partial complex I loss restricted to some areas, it is possible that mutations cannot be detected.

Potentially pathogenic mutations were found in 3.8% (3/78) diffuse carcinomas, including one stop mutation and two missense mutations. The following 37 nuclear complex I genes were checked for mutations: NDUFA1, 2, 3, 5, 6–13; NDUFAB1; NDUFB1–11; NDUFC1–2; NDUFS1–8; NDUFV1–3. In summary, a 2.5-fold higher frequency of nuclear complex I mutations in intestinal carcinomas compared to diffuse carcinomas was found.

### 3.5. VacA Positivity Does Not Correlate with OXPHOS Alterations

As indicated in the literature, the major HP virulence factor VacA seems to affect mitochondrial biology. To investigate if HP infection, which is a known risk factor for the development of intestinal GC, influences mitochondrial biology in gastric cells, we immunohistochemically analyzed biopsies from type B gastritis with and without metaplasia, type C gastritis, and GC patients for the presence of the major virulence factor VacA by immunohistochemical staining. We also examined the expression of complex I, mitochondrial transcription factor A (TFAM), and porin as markers for mitochondrial biogenesis.

**Figure 6:**
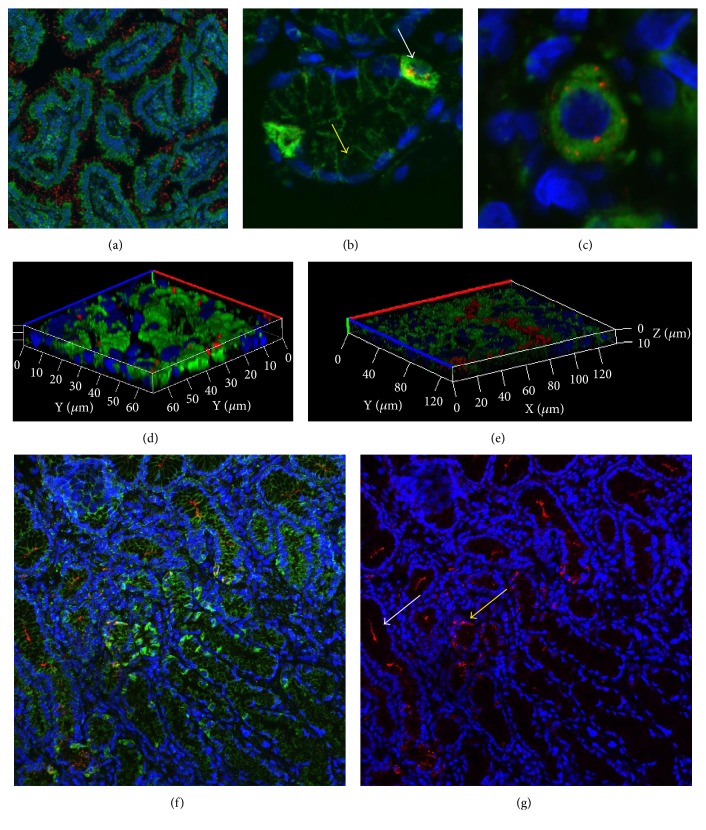
Localization of VacA in gastritis. (a) Triple immunofluorescence staining (complex I/VacA/DAPI) of case BGM-4 revealed strong HP colonization of the gastric lumen (10x magnification). (b) Triple immunofluorescence staining (porin/VacA/DAPI) of case BG-3 revealed selective staining of mitochondria-rich parietal cells. No differences in porin expression between the infected and noninfected parietal cells were detected. Parietal cells (white arrow) and mucous-secreting cells (yellow arrow) (63x magnification). (c) Triple immunofluorescence staining (TFAM/VacA/DAPI) of case BG-3 showed intracellular staining of VacA in parietal cells. (d) Z-stack of triple immunofluorescence staining (TFAM/VacA/DAPI) of case BG-1 revealed cytoplasmic localization of VacA (63x magnification). (e) Intraglandular VacA in case BG-1 (TFAM/VacA/DAPI); (20x magnification). (f) Triple immunofluorescence staining (Porin/VacA/DAPI) of case BG-3. (g) Figure f without the green channel to better visualize the intraglandular (white arrow) and intraparietal (yellow arrow) localization of VacA.

**Figure 7:**
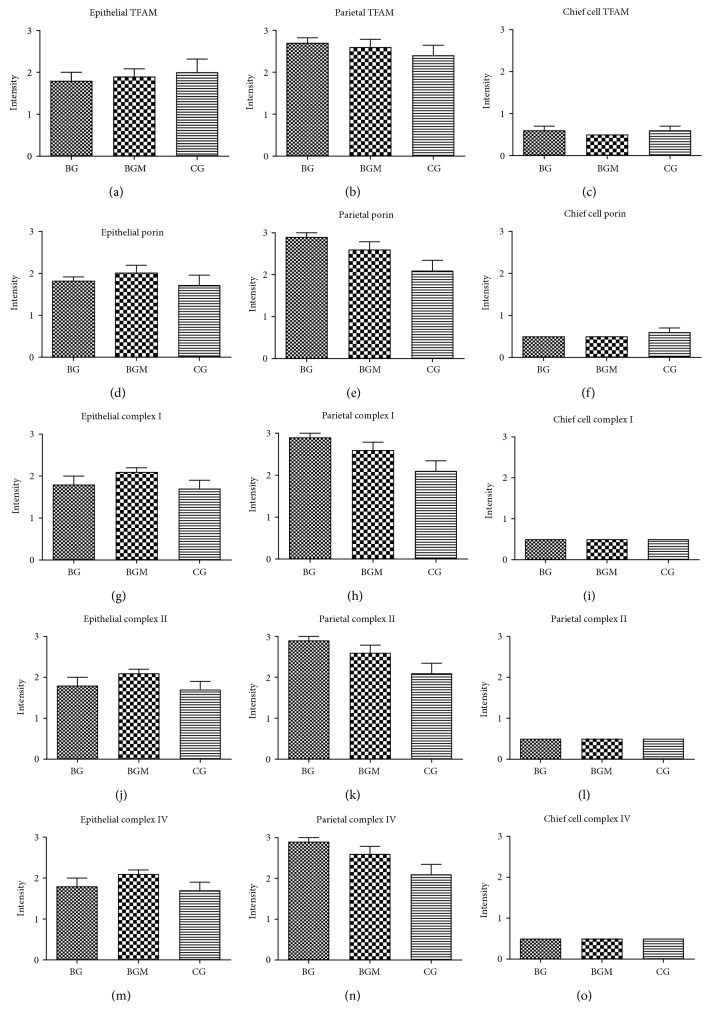
No differences of the OXPHOS complex, porin, and TFAM expression between chemical, bacterial, and bacterial gastritis with metaplasia. (a–c) TFAM expression in bacterial gastritis (BG), bacterial gastritis with metaplasia (BGM), and chemical gastritis (CG). (d–f) Porin expression. (g–i) Complex I expression. (j–l) Complex II expression. (m–o) Complex IV expression. (a), (d), (g), (j), and (m) epithelial cells; (b), (e), (h), (k), and (n) parietal cells; (c), (f), (i), (l), and (o) chief cells. For statistical analysis, a nonparametric Kruskal-Wallis test was used. Note that in some figures, no SEM is indicated because the staining intensity was the same for all samples. For all groups *n* = 5.

VacA was never detected in type C gastritis. We detected VacA mainly in gastric glands (Figures 6(f) and 6(g)) and the gastric lumen (Figure 6(a)), consistent with its localization in previous studies [38, 47]. Chief cells never showed positivity for VacA, and intracellular VacA was rarely detected in epithelial cells. Interestingly, we observed localization of VacA to human gut parietal cells in biopsies of patients with HP gastritis (Figures 6(b), 6(c), 6(d), 6(f), and 6(g) and Figure 7). HP is able to repopulate the extracellular gut environment after complete elimination of extracellular bacteria with gentamicin [48, 49], suggesting that HP may be sheltered for long periods within some host cell population, like parietal cells.

Parietal cells are mitochondria-rich whereas chief cells only show weak porin expression (Figures 6(b) and 6(f) and Figures 2(g), 2(h), and 2(i)).

We found no correlation between VacA positivity and TFAM or porin staining intensity in gastritis (Figures 6 and Figure 7). The chemical (*n* = 5), bacterial (*n* = 5), and bacterial gastritis cases with metaplasia (*n* = 5) did not differ in terms of expression of TFAM, porin, and the OXPHOS complexes (Figure 7). In general, expression of all mitochondrial markers was significantly lower in chief cells than in epithelial and parietal cells (Figure 7), as expected.

## 4. Discussion

About one third (30%) of the 20 intestinal GCs we examined exhibited focal complex I deficiency, with 35–65% of the tumor cells negative for complex I. It was reported that gastric cancers are composed of different tumor cell clones [50, 51]. However, few OXPHOS-deficient tumors might be overseen by immunohistochemical analysis, since it is proposed that mutations just affecting the enzymatic activity and not protein levels exist, but this might be the exception not the rule.

In only one of these six cases, the complex I deficiency could be explained by a pathogenic mtDNA mutation. The relatively low percentage of mitochondrial DNA mutations in our sample cohort (5%) points to a limited significance; Habano et al. also reported potentially pathogenic mitochondrial complex I mutations in 2/34 (6%) of their analyzed intestinal carcinomas [19]. As most of the complex I subunits are encoded by nuclear genes, mutations in the latter are likely responsible for the isolated loss of complex I in most cases of intestinal GC. In summary, a 2.5-fold higher frequency of nuclear complex I mutations in intestinal carcinomas compared to diffused carcinomas was found by analysis of data from the Cancer Genome Project suggesting that indeed nuclear mutations might contribute to the observed complex I loss in a high percentage of intestinal carcinomas. The phenomenon that complex I is most frequently affected has several reasons. First, complex I is the biggest OXPHOS complex composed of 45 (44 + 1) subunits. Therefore, more mutations might be found in complex I since 44 genes encode complex I subunits but only 4 genes encode for complex II subunits. In addition, mitochondrial DNA is specifically prone to damage. Complex II is encoded by nuclear genes. Second, complex I is linked to several other important metabolic pathways and apoptosis. Mitochondrial fatty acid biosynthetic pathway and lipoylation important especially for the pyruvate dehydrogenase are linked to the acyl carrier protein (ACP; NDUFAB1) [52]. Defects of complex I have pleiotropic effects leading to more global changes and reprogramming of cancer metabolism. Furthermore, the complex I subunit NDUFS1 can be cleaved by caspase 3 and granzyme A to induce apoptosis [53, 54]. Therefore, lack of complex I might represent a growth advantage for tumor cells.

In the 20 cases of diffuse-type GC that we examined, complex I deficiency was rare. Therefore, complex I deficiency might be of importance for the pathogenesis of intestinal GC only. However, as we found no complex I-negative cells in type B gastritis with or without metaplasia or in type C gastritis, loss of complex I does not appear to be an early event in GC tumor development. Moreover, HP-infected cells (parietal cells, mucous-secreting cells, and metaplasia) showed no signs of OXPHOS dysregulation.

In complex I-deficient tumors, compensatory mechanisms are found which indicate that a partial rescue of mitochondrial respiration can be achieved by an increase of mitochondrial biogenesis, especially complex II [55]. Since complex II is able to feed electrons into the respiratory chain, it is thought that residual respiration can be sustained via complex II in complex I-deficient tumor cells. However, complex II is not able to transport protons across the mitochondrial membrane.

Comparison of the seven cases with OXPHOS deficiencies revealed that porin, the marker for the mitochondrial mass, is higher in the OXPHOS-deficient cases compared to OXPHOS competent cases.

Also other proteins feed electrons into the respiratory chain like the mitochondrial sulfide:quinone oxidoreductase (SQRDL), the electron transfer flavoprotein-ubiquinone oxidoreductase (ETFDH), the glycerol-3-phosphate dehydrogenase (GPD2), and the sulfite oxidase (SUOX) that potentially contribute to residual respiration in complex I-negative tumors [56–59]. However, evidence that mitochondrial respiration is crucial for tumor cells comes from therapeutic studies, which, for example, show that inhibition of complex I and glucose restriction is lethal for cancer cells [60].

Karita et al. reported VacA localization in mucous neck, chief, and parietal cells of HP-infected patients [38]. However, to our knowledge, ours is the first study to show VacA positivity exclusively in parietal cells. We detected intracellular localization of this major pathogenicity factor of HP in parietal cells but found no positivity in chief cells. In addition, we noted obvious VacA/HP positivity in the lumen of gastric glands and the gastric lumen. VacA can permeabilize the apical membrane of isolated rat parietal cells, inducing hypochloridia [61]. The selective localization in parietal cells has potential therapeutic implications, because HP might avoid drug therapy by localizing in deep layers of the mucosa [38, 62, 63]. HP can repopulate the extracellular gut environment after complete elimination of extracellular bacteria with gentamicin [49, 64].

Importantly, in our study, intestinal GCs in patients with a history of HP infection had a lower mitochondrial mass compared to cases with no prior HP infection, as indicated by porin expression. Since the OXPHOS complexes showed a trend (complex III significantly reduced) toward lower levels in cases with an HP-positive history, HP might therefore affect mitochondrial function only in intestinal GC cells, since we found no alterations of mitochondrial parameters in any of the gastritis cases. We speculate that some mitochondrial alterations reported in the literature might be associated with induction of apoptosis and not HP infection per se as previously reported [36]. We suppose that results have to be interpreted with regard to acute or chronic infection. It is highly doubtful that the high MOIs (100–400) which are usually used for cell culture experiments would be reached during infection in vivo [65, 66]. Previous studies supposed that MOIs between 1 and 10 are found in the stomach [65, 66]. Low MOIs (1–10) inhibit apoptosis whereas high MOIs (>75) induce apoptosis in splenocytes [67].

Previous studies reported that infection with low MOI (=10) did not induce mtDNA destabilization [35]. We suggest that studies that used a very high MOI are of limited biological significance, because most of the reported changes in mitochondrial energy metabolism might be attributable to apoptotic changes. Furthermore, based on molecular genetic analysis, mainly, mutations in the highly variable D-loop region of mtDNA were found, alterations unlikely to cause severe mitochondrial dysfunction [34, 68].

However, the biological significance seems to be limited, as we found no associations between infection and parameters of mitochondrial energy metabolism in gastritis. Our data indicate that the alterations of energy metabolism found in a large percentage of GCs might occur independently of bacterial infection.

Although HP can influence mitochondrial physiology in vitro, there are no signs of impact on the OXPHOS system in gastritis and cancer. A high percentage of intestinal gastric carcinomas exhibits complex I deficiency, independent of HP history. Complex I deficiency might have therapeutic implications since tumors with mitochondrial defects and high glycolytic activity can potentially be inhibited in growth via a low-carbohydrate, high-fat (ketogenic) diet.

Evidence suggests that HP can overcome eradication therapy and repopulate the gastric lumen from deep layers of the mucosa. Intracellular *Helicobacter pylori* VacA positivity is found exclusively in parietal cells that might be used as a niche. Testing for HP positivity especially VacA in deep layers might be important for evaluation of the effectiveness of eradication therapy.

In addition, correlations between OXPHOS complex expression and parameters of tumor malignancy were present. A significantly higher complex I and complex II expression was present in large tumors. Tumor size is an independent prognostic factor for a 5-year survival rate in advanced gastric cancer [69, 70]. The mean tumor size in the study by Wang et al. was 4.9 cm who analyzed 430 individuals. In our study, the mean size was 5 cm. Furthermore, a higher expression of complex II was found in grade 3 versus grade 2 and T4 versus T1 gastric carcinomas. The higher complex II levels found in high grade tumors might reflect the degree of OXPHOS deficiency. A highly dysfunctional respiratory chain might lead to a stronger compensatory upregulation of complex II to sustain a residual OXPHOS. Significantly, higher complex III expression was also found in UICC III compared to UICC II tumors. Significantly lower complex III protein levels were found in tumors localized in the prepyloric antrum compared to the cardia (*p* = 0.049). This is consistent with the previous results. Patients with tumors of the antrum show a better survival than individuals with tumors of the antrum [71]. The more malignant tumors therefore again show a higher OXPHOS enzyme expression. However, it has to be mentioned that the increased expression of OXPHOS complexes might be due to a compensatory effect. Patients with a lack of complex I frequently show an increase of the other respiratory chain complexes. A high expression of complex II does not exclude complex I deficiency; rather, it could be suggestive of a respiratory chain defect. However, in case of our study, no compensatory upregulation of complex II was present in the 35% of the intestinal carcinomas with complex I deficiency. Complex II and complex III were higher in the tumors without complex I deficiency. An increase in complex II might cause an increase in respiration, since complex II can feed electrons into the transport chain. A singular upregulation of complex III should not be sufficient to enhance OXPHOS. In addition, the mitochondrial mass was equal in both groups suggesting no compensatory mechanism. In summary, gastric carcinomas exhibit a high percentage of OXPHOS enzyme defects and an association of a high expression of several OXPHOS enzymes and malignancy.

## Supplementary Material

Supplementary figure 1: (A) Complex II in normal adjacent tissue. (B) Complex II in intestinal gastric carcinoma (C) Complex V in normal adjacent tissue. (D) Complex V in tumor tissue. (Case M14). In A and C again parietal cells are visible (dark brown staining). Magnification 10x. Supplementary figure 2: Immunohistochemical staining of the OXPHOS complexes of normal tissue. (A-C; G-I) Diffuse gastric carcinoma; (D-F; J-L) Tumor tissue. (A,D) Porin; (B,E) complex I; (C,F) complex II; (G,J) complex III; (H,K) complex IV; (I,L) Complex V. (Case M39) Magnification 10x. Supplementary table 1: Primers used for amplification and sequencing of mtDNA. Supplementary table 2: Clinical charateristics including tumor classification. Supplementary table 3: Influence of clinico-pathological parameters on expression of mitochondrial proteins. Supplementary table 4 - Part F Score values for complex V in intestinal gastric carcinoma. Supplementary table 5 - Part F Score values for complex V in diffuse gastric carcinoma.
