# Supplementary material for: Oxidative Phosphorylation System in Gastric Carcinomas and Gastritis
Source: Oxid Med Cell Longev. 2017 Jun 28;2017:1320241. doi: 10.1155/2017/1320241 (PMC5506471; doi:10.1155/2017/1320241)
Supplement: Supplementary file 1 — Supplementary figure 1: (A) Complex II in normal adjacent tissue. (B) Complex II in intestinal gastric carcinoma (C) Complex V in normal adjacent tissue. (D) Complex V in tumor tissue. (Case M14). In A and C again parietal cells are visible (dark brown staining). Magnification 10x. Supplementary figure 2: Immunohistochemical staining of the OXPHOS complexes of normal tissue. (A-C; G-I) Diffuse gastric carcinoma; (D-F; J-L) Tumor tissue. (A,D) Porin; (B,E) complex I; (C,F) complex II; (G,J) complex III; (H,K) complex IV; (I,L) Complex V. (Case M39) Magnification 10x. Supplementary table 1: Primers used for amplification and sequencing of mtDNA. Supplementary table 2: Clinical charateristics including tumor classification. Supplementary table 3: Influence of clinico-pathological parameters on expression of mitochondrial proteins. Supplementary table 4 - Part F Score values for complex V in intestinal gastric carcinoma. Supplementary table 5 - Part F Score values for complex V in diffuse gastric carcinoma. [file 1320241.f1.pptx]

## Slide 1
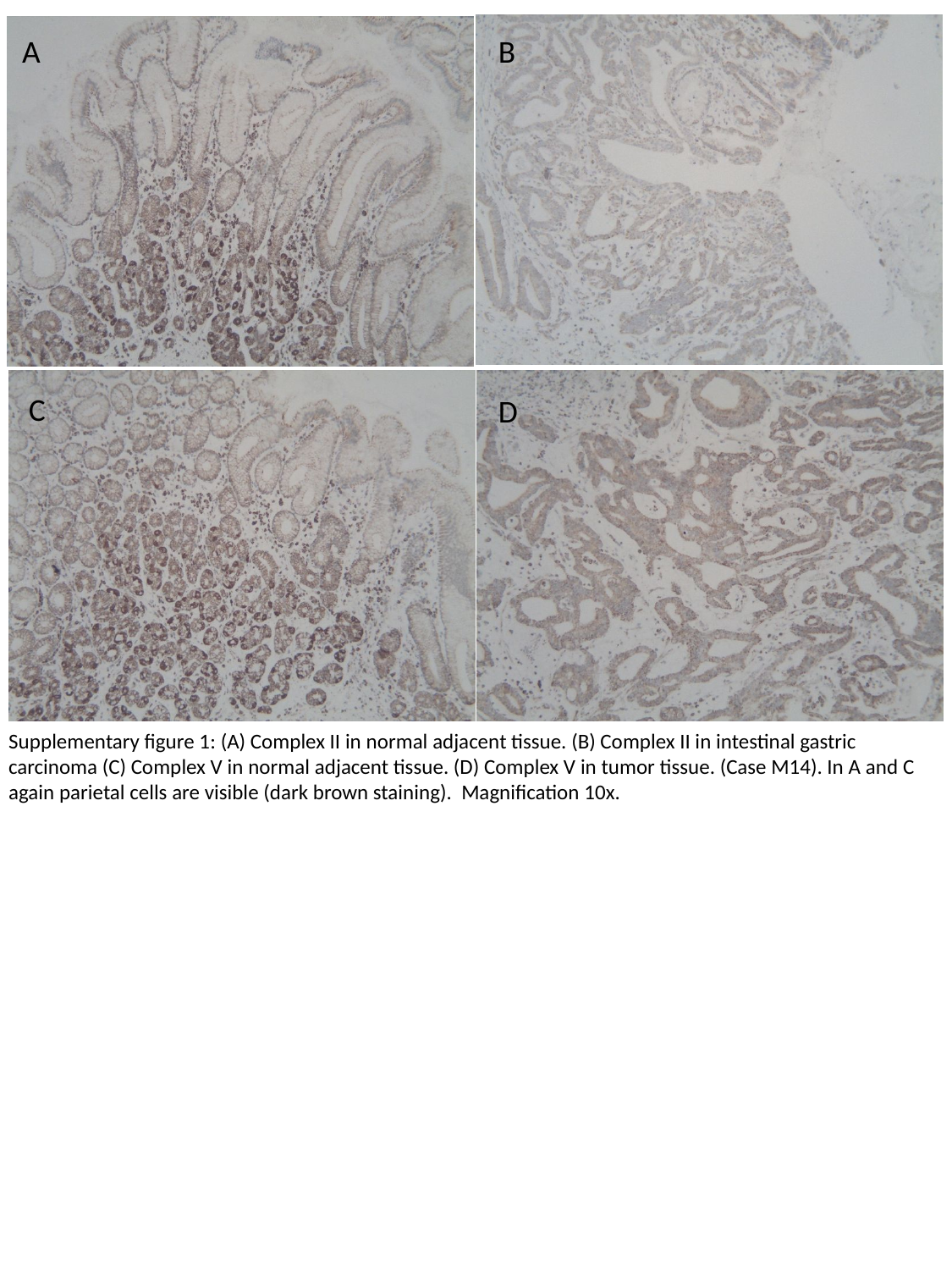

A
B
C
D
Supplementary figure 1: (A) Complex II in normal adjacent tissue. (B) Complex II in intestinal gastric carcinoma (C) Complex V in normal adjacent tissue. (D) Complex V in tumor tissue. (Case M14). In A and C again parietal cells are visible (dark brown staining). Magnification 10x.
