# Supplementary material for: Oxidative Phosphorylation System in Gastric Carcinomas and Gastritis
Source: Oxid Med Cell Longev. 2017 Jun 28;2017:1320241. doi: 10.1155/2017/1320241 (PMC5506471; doi:10.1155/2017/1320241)
Supplement: Supplementary file 2 [file 1320241.f2.pptx]

## Slide 1
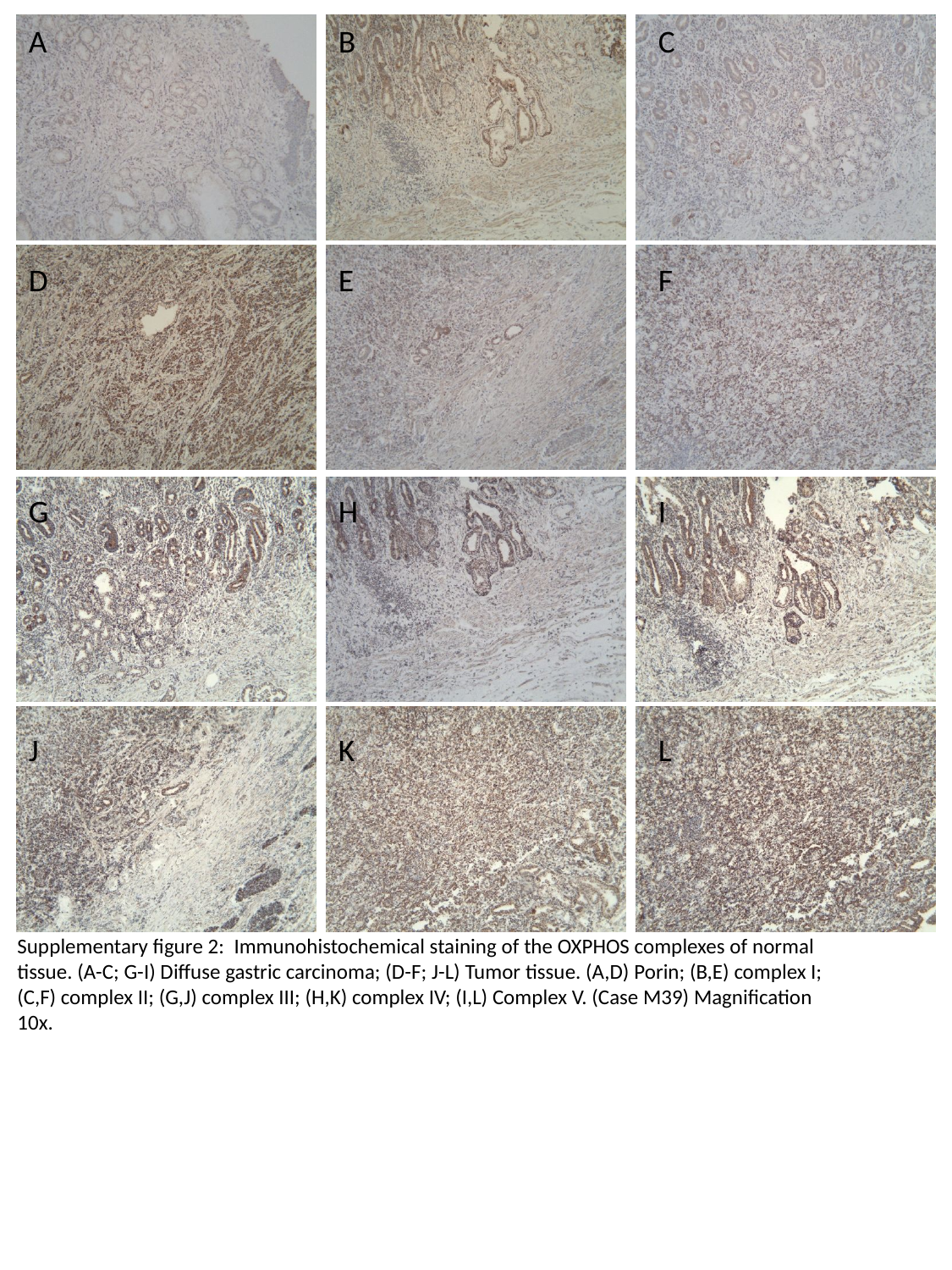

A
B
C
D
E
F
G
H
I
J
K
L
Supplementary figure 2: Immunohistochemical staining of the OXPHOS complexes of normal tissue. (A-C; G-I) Diffuse gastric carcinoma; (D-F; J-L) Tumor tissue. (A,D) Porin; (B,E) complex I; (C,F) complex II; (G,J) complex III; (H,K) complex IV; (I,L) Complex V. (Case M39) Magnification 10x.
