# Supplementary material for: Oxidative Phosphorylation System in Gastric Carcinomas and Gastritis
Source: Oxid Med Cell Longev. 2017 Jun 28;2017:1320241. doi: 10.1155/2017/1320241 (PMC5506471; doi:10.1155/2017/1320241)
Supplement: Supplementary file 4 [file 1320241.f4.docx]

**Supplementary table 2: Clinical charateristics including tumor classification**

| **Category**  **n (%)** | **All gastric cancer specimens** | *Intestinal cancer* | *Diffuse Cancer* | p |
| --- | --- | --- | --- | --- |
| **N^1^**  male /female (%) | 40 (100)  18 (45)/22 (55) | 20 (50)  10 (50)/10 (50) | 20 (50)  8 (40)/12 (60) | n.s. |
| **Age^2^** mean years (95% CI) | 70.1 (66.8-73.4) | 73.8 (70.1 - 77.5) | 66.5 (61.2 - 71.7) | ***** |
| **Tumor localisation^1^**  (ca/co/iz/an/pa)^a^ | 5/12/6/12/5 | 1/8/2/5/4 | 4/4/4/7/1 | n.s. |
| **Tumor size^2^**: mean cm (95% CI) | 5.0 (3.8-6.2) | 4.0 (2.9-5.1) | 6.2 (3.8-8.5) |  |
| **Grading^1^**   - **1** - **2** - **3** | 1  11  28 | 1  10  9 | 0  1  19 | ****** |
| **T staging^1^**   - **1 : 1a/1b** - **2** - **3** - **4: 4a/4b** | 9: 2/7  6  10  15: 12/3 | 6: 1/5  3  7  4: 3/1 | 3: 1/2  3  3  11: 9/2 | n.s. |
| **N status^1^**   - **0** - **1** - **2** - **3: 3a/3b** | 16  8  3  13: 4/9 | 10  6  2  2: 0/2 | 6  2  1  11: 4/7 | ***** |
| **M status^1^**   - **0** - **1** | 36  4 | 19  1 | 17  3 | n.s. |
| **UICC^1^**   - **I : IA/IB** - **II: IIA/IIB** - **III: IIIA/IIIB/IIIC** - **IV** | 12: 8/4  10: 6/4  14: 2/4/8  4 | 6: 5/1  10: 6/4  3: 0/1/2  1 | 6: 3/3  0  11: 2/3/6  3 | ***** |
| **R status^1^**   - **0** - **1** - **2** | 33  6  1 | 17  2  1 | 16  4  0 | n.s. |
| **Survival^1/2^**   - No/yes - Mean months (95% CI) - Median months (min-max) | 35/5  47.4 (31.1-63.7)  34.3 (1.0-123.2) | 17/3  45.9 (26.1-65.7)  51.9 (1.0-117.8) | 18/2  49.5 (17.7-81.3)  21.0 (2.3-123.2) | n.s. |

Tumor-classification is performed according the 7th TNM (see Wittekind C, Meyer HJ (2010) TNM - Klassifikation maligner Tumoren, 7th Edition. Wiley-VCH: Weinheim, Germany, ISBN: 978-3-527-66949-3). Applied statistics: ^1^χ^2^ test, ^2^Student´s t-test with following significance levels: *p <0.05, **p <0.01; ^a^ca = cardia, co = corpus, iz = intermediate zone, an = antrum, pa = prepyloric antrum.
