# Supplementary material for: Oxidative Phosphorylation System in Gastric Carcinomas and Gastritis
Source: Oxid Med Cell Longev. 2017 Jun 28;2017:1320241. doi: 10.1155/2017/1320241 (PMC5506471; doi:10.1155/2017/1320241)
Supplement: Supplementary file 5 [file 1320241.f5.docx]

**Supplementary table 3: Influence of clinico-pathological parameters on expression of mitochondrial proteins.**

| **Parameter**  (appplied categories) | **Complex 1** | **Complex 2** | **Complex 3** | **Complex 4** | **Complex 5** | **Porin** |
| --- | --- | --- | --- | --- | --- | --- |
| **Gender**^1^  (male /female) | n.s. | n.s. | n.s. | n.s. | n.s. | n.s. |
| **Age**^1^  (low/high)^a^ | n.s. | n.s. | n.s. | n.s. | n.s. | n.s. |
| **Tumor localisation**^2^  (ca/co/iz/an/pa)^b^ | n.s. | n.s. | ca (265.5) > pa (148.5)  p = 0.049 | n.s. | n.s. | n.s. |
| **Tumor size**^1^  (low/high) ^a^ | Low (114.9) < High (163.7)  p = 0.005 | Low (104.3) < High (162.0)  p = 0.011 |  | n.s. | n.s. | n.s. |
| **Grading**^1^  (2-3) | n.s. | G2 (84.0) < G3 (139.1)  p = 0.018 | G2 (162.2) < G3 (219.6)  p = 0.016 | n.s. | n.s. | n.s. |
| **T staging**^2^  (1-4) | n.s. | T1 (73.0) < T4 (157.8)  p = 0.013 | n.s. | n.s. | n.s. | n.s. |
| **N status**^2^  (0-4) | n.s. | n.s. | n.s. | n.s. | n.s. | n.s. |
| **M status**^1^  (0-1) | n.s. | n.s. | n.s. | n.s. | n.s. | n.s. |
| **UICC**^2^  (1-4) | n.s. | n.s. | UICC II (155.2) < III (232.3)  p = 0.030 | n.s. | n.s. | n.s. |
| **R status**^1^  (0-1) | n.s. | n.s. | n.s. | n.s. | n.s. | n.s. |
| **Survival**^1^  (yes/no) | n.s. | n.s. | n.s. | n.s. | n.s. | n.s. |

Tumor-classification is performed according the 7th TNM (see Wittekind C, Meyer HJ (2010) TNM - Klassifikation maligner Tumoren, 7th Edition. Wiley-VCH: Weinheim, Germany, ISBN: 978-3-527-66949-3). Applied statistics: ^1^Student´s t-test, ^2^ANOVA (Bonferroni post-hoc test). ^a^The parameter age and tumor size were separated into low and high case by the mean age (of 70.1 [years]) and mean tumor size (of 5.0 [cm]), respectively. ^b^ca = cardia, co = corpus, iz = intermediate zone, an = antrum, pa = prepyloric antrum. Values given in brackets indicate score values of the analysis of the immunohistochemical staining.
